# Extensive Secondary Cratering From the InSight Sol 1034a Impact Event

**DOI:** 10.1029/2024JE008535

**Published:** 2024-12-18

**Authors:** P. M. Grindrod, I. J. Daubar, B. Fernando, D. Kim, G. S. Collins, S. C. Stähler, N. Wojcicka, L. V. Posiolova, M. Froment, É. Beucler, E. Sansom, R. Garcia, G. Zenhäusern

**Affiliations:** ^1^ Natural History Museum London UK; ^2^ Department of Earth Environmental and Planetary Sciences Brown University Providence RI USA; ^3^ Department of Earth and Planetary Sciences Johns Hopkins University Baltimore MD USA; ^4^ Department of Earth Science and Engineering Imperial College London London UK; ^5^ Institute of Geophysics, ETH Zurich Zurich Switzerland; ^6^ Malin Space Science Systems San Diego CA USA; ^7^ École Normale Supérieure Paris‐Saclay Cachan France; ^8^ Earth and Environmental Sciences Division Los Alamos National Laboratory Los Alamos NM USA; ^9^ Laboratoire de Planétologie et Géodynamique Université de Nantes Nantes France; ^10^ International Centre for Radio Astronomy Research Curtin University Perth WA Australia; ^11^ Space Science and Technology Centre Curtin University Perth WA Australia; ^12^ Institut Supérieur de l'Aéronautique et de l'Espace SUPAERO Toulouse France; ^13^ Institut für Geophysik, ETH Zurich Zurich Switzerland

**Keywords:** Mars, crater, seismic, InSight, secondary, impact

## Abstract

Impact cratering is one of the fundamental processes throughout the history of the Solar System. The formation of new impact craters on planetary bodies has been observed with repeat images from orbiting satellites. However, the time gap between images is often large enough to preclude detailed analysis of smaller‐scale features such as secondary impact craters, which are often removed or buried over a short time period. Here we use a seismic event detected on Mars by the NASA InSight mission to investigate secondary cratering at a new impact crater. We strengthen the case that the seismic event that occurred on Sol 1034 (S1034a) is the result of a new impact cratering event. Using the exact timing of this event from InSight, we investigated the resulting new impact crater in orbital image data. The S1034a impact crater is approximately 9 m in diameter but is responsible for over 900 secondary impact events in the form of low albedo spots that are located at distances of up to almost 7 km from the primary crater. We suggest that the low albedo spots formed from relatively low energy ejecta, with individual ejecta block velocities less than 200 m s^−1^. We estimate that the low albedo spots, the main evidence of secondary impact processes at this new impact event, fade within 200–300 days after formation.

## Key Points

- A new 9‐m‐diameter impact crater on Mars identified with in situ seismic and orbital image data
- Over 900 low albedo spots identified as a result of secondary ejecta processes
- Small impact events have likely been captured in existing data sets and not yet recognized

## 1 Introduction

Impact cratering is a ubiquitous process throughout the Solar System. The formation rate of impact craters on planetary surfaces (the “production function”) has evolved over time (e.g., Hartmann & Neukum, 2001; Neukum et al., 2001), with this flux a key parameter in determining crater retention model surface ages (Hartmann et al., 2007). In the absence of returned samples, using crater size‐frequencies to estimate the approximate age of a planetary surface is a vital, and commonly used, tool in planetary science (e.g., Robbins, 2014). The recent increase in spatial and temporal resolution of orbiting imaging instruments has allowed the refinement of the present‐day bombardment rate on Mars (Daubar et al., 2013) and the Moon (Speyerer et al., 2016), and an improvement in the crater counting method.

The observation of new impact crater‐forming events from orbital data is a relatively recent development. Using the Mars Orbiter Camera on the Mars Global Surveyor spacecraft, Malin et al. (2006) identified 20 new impact sites, and a further 24 candidate sites, over a 7 year period. By 2012, using a combination of candidate impact site discovery with Context Camera (CTX; Malin et al., 2007) images, followed by impact site confirmation with High Resolution Imaging Science Experiment (HiRISE; McEwen et al., 2007) images, Daubar et al. (2013) cataloged 248 new impact sites that occurred over the previous two decades. Continued observation has led to the most recent catalog of new impact sites on Mars, which contains 1203 confirmed impact craters, ranging from 1 to 58 m in diameter (Daubar et al., 2022). One of the scientific goals of the NASA Interior Exploration using Seismic Investigations, Geodesy and Heat Transport (InSight) mission at Mars was to attempt to detect the seismic signals of meteoroid impacts at the surface (Banerdt et al., 2020). After no seismic detections of impact cratering events were identified during the first martian year of surface operations, InSight data has now been used to detect 8 impact events (Daubar et al., 2023, 2024), all confirmed with orbital imaging, up to 150 m in diameter (Posiolova et al., 2022), and with several occurring in clusters (Garcia et al., 2022). Together, these orbital and in situ seismic studies have helped to refine the present‐day impact flux on Mars.

**Figure 1:**
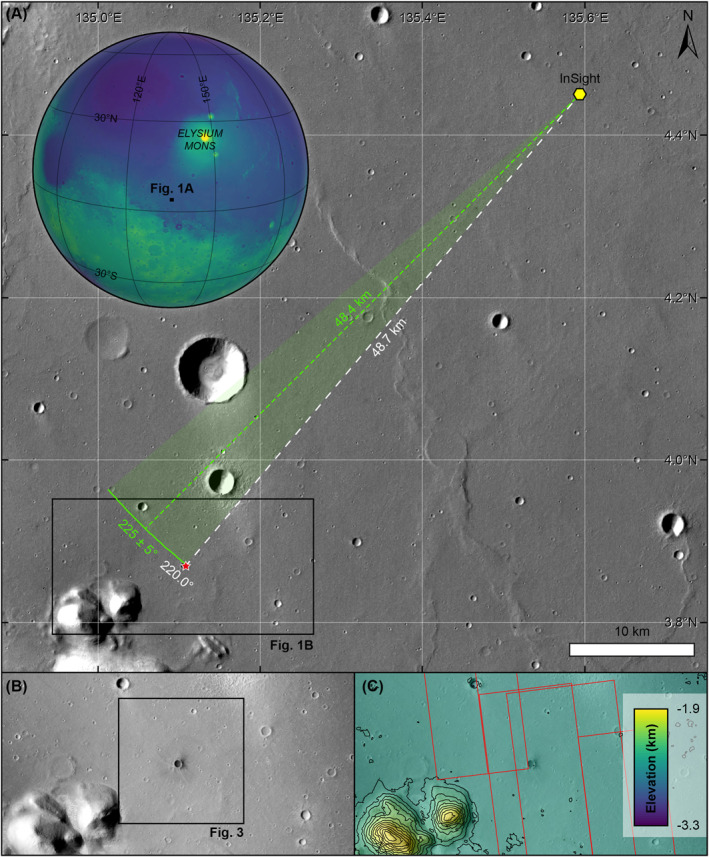
The S1034a study region. (a) The context of the study region. Inset shows the location of (a) on Mars with a colorized Mars Orbiter Laser Altimeter globe (blues are low elevation, green and yellows are higher elevation). The main image is HRSC image hh852_0009_nd3, with the location of the InSight lander (yellow hexagon) and S1034a impact event (red star) indicated. Dashed green line shows the event distance estimated from seismic analysis. Solid green line and semi‐transparent green area show the back azimuth estimate and error, respectively, from seismic analysis. The black box shows the location of (b). (b) The local study region, as seen in CTX image U03_072288_1821_XI_02N224W, taken 66 days after the impact event. The black box shows the location of Figure 3. (c) Elevation of the local study region from a CTX stereo Digital Terrain Model (DTM). Contours are every 100 m. Red polygons show the location of HiRISE images used in this study (Table 1).

In terms of quantifying the longer‐term impact flux on Mars, the relative contribution of secondary versus primary impact craters, particularly at small sizes, is a topic of ongoing debate (e.g., Bierhaus et al., 2018; McEwen & Bierhaus, 2006; McEwen et al., 2005; Neukum & Ivanov, 1994). One aspect of this problem is that we lack an accurate understanding of the number, extent, and spatial distribution of secondary craters produced from a single primary impact event. In this study, we address this issue by studying a new impact event on Mars (Figure 1), detected by the InSight mission, and observed with orbital assets. We used visible wavelength images from different instruments and missions, including the NASA Mars Reconnaissance Orbiter CTX and HiRISE instruments, and the European Space Agency (ESA) ExoMars Trace Gas Orbiter Color and Stereo Surface Imaging System (CaSSIS (Thomas et al., 2017)). We focus our study on the S1034a seismic event as recorded by the Seismic Experiment for Interior Structure (SEIS) instrument (Lognonné et al., 2019) on InSight, and the number and distribution of secondary craters in the surrounding area. This particular event was detected seismically by InSight, but at the time no corresponding location event was identified (Garcia et al., 2022), and thus without in situ assets would likely have been excluded from orbital catalogs. Our goal is twofold: (a) to provide new insights into detecting impact events on Mars, and (b) to provide new insights into secondary impact processes. We first describe the nature of the seismic event, and its wider importance in the Mars Seismic Catalog (Ceylan et al., 2022, 2023), before detailing the nature of the new impact event, and the implications of secondary impact processes during this event.

## 2 InSight Sol 1034 Seismic Event

**Figure 2:**
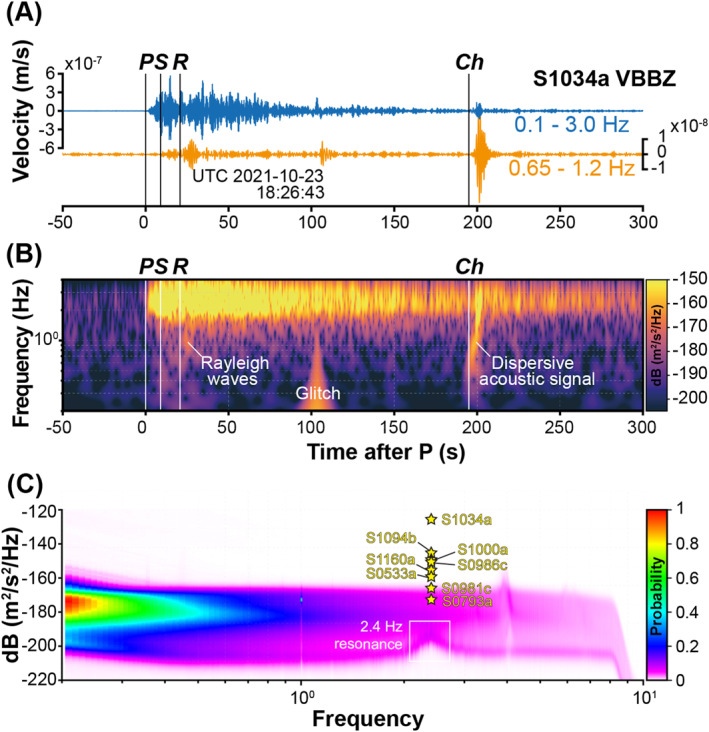
Seismic recording of S1034a. (a) Very broadband (VBB) vertical‐component seismic waveform of S1034a band‐pass filtered between 0.1 and 3.0 Hz (blue) and 0.65–1.2 Hz (orange). (b) Vertical‐component S‐transform of S1034a. Arrival times of P‐ and S‐wave (P & S; InSight Marsquake, 2023), surface wave (R; Drilleau et al., 2023), glitch (Ceylan et al., 2022; Kim et al., 2021), and chirp (Ch; Garcia et al., 2022) signals are indicated by vertical lines. (c) Ground velocity spectral density measured in the peak energy at 2.4 Hz using VBBZ data of confirmed impact events (yellow stars). Background noise statistics are based on 2019–2022 recordings of VBBZ during the InSight mission.

Event S1034a occurred at 18:26:30 UTC on 2021‐10‐23, with a duration of approximately 6–7 min (Figure 2). Categorized as Very High‐frequency (VF) based on its spectral content, this event occurred in close proximity to the InSight lander within a distance of less than 1°. For the high‐frequency family of events including VF events on Mars, the Marsquake Service (MQS; InSight Marsquake (2023)) determines the Mars moment magnitude $\left(M_{W}^{Ma}\right)$ by calibrating a magnitude scale derived from measuring the amplitude of the peak energy at 2.4 Hz (Böse et al., 2021). This resulted in an original estimate of $M_{W}^{Ma}$ 3.0 ± 0.2 for the S1034a event, making it the largest among the confirmed nearby impacts to date (Daubar et al., 2023; Garcia et al., 2022). Our understanding of the true nature of the 2.4 Hz resonance in the continuous seismic recordings on Mars (Dahmen et al., 2021; Hobiger et al., 2021) and its amplification during a seismic event is limited. This limitation poses the potential for overestimating moment magnitudes in VF events, and as a result, the magnitude of this event was recently refined to 2.1, with the close proximity and interactions with subsurface structure responsible for the apparent amplification (Zenhäusern et al., 2024).

Notably, a chirp (a short dispersive signal with frequency increasing over time) is observed approximately 200 s after the main P‐wave arrival. This dispersion is typical of guided acoustic waves, likely stemming from an impact source (Garcia et al., 2022). The polarization of this acoustic signal, in addition to those from the P‐ and S‐waves, allows more refined determination of the back azimuth to the source toward the south‐west of the lander. Recently, surface waves were identified in this event at a relatively high frequency near 1 Hz using a signal coherency approach (Drilleau et al., 2023). A comparable, albeit much weaker signal, was also observed in the S0986c impact. Combined with preceding discoveries in two distant large impacts, S1094b and S1000a (Kim et al., 2022), the presence of surface waves is a distinctive characteristic observed in confirmed impact recordings within the InSight seismic data, distinguishing them from most events originating from tectonic activity (e.g., Stähler et al., 2022).

## 3 Candidate New Impact Crater

Unlike other meteoroid impacts detected on Mars by InSight (e.g., Posiolova et al., 2022), the location of the candidate impact crater for the S1034a event precluded discovery in CTX image searches, and was instead first identified indirectly in a HiRISE image (ESP_073633_1840). Low albedo spots with a directional “tail” up to approximately 15 m in length were discovered, and had a collective orientation that suggested an origin that was centered in an area outside of the HiRISE image. Analysis of a CaSSIS image (MY36_019001_177) expanded the number of low albedo spots, while also refining the timing of the impact event, and the likely location of the primary impact crater. Reanalysis of CTX images allowed further refinement of the timing, and the determination of the likely impact crater before confirmation with new HiRISE images (ESP_074701_1840, ESP_075901_1840). Subsequent imaging has allowed a more extensive analysis of the entire impact effects, which are described below. The timing, instrument source, and spatial resolution of each image used in this study are given in Table 1.

**Table 1:** Images Used in the Search and Analysis of the S1034a Event

Instrument	ID	Date	Timing	Feature	Resolution (m/px)
CTX	D10_031157_1845_XN_04N225W	2013‐03‐20	Before	–	6.56
CTX	N17_069097_1848_XI_04N225W	2021‐04‐23	Before	–	5.42
CTX	N22_071075_1861_XN_06N225W	2021‐09‐24	Before	–	5.38
CTX	U03_072288_1821_XI_02N224W	2021‐12‐28	After	Crater	5.51
CTX	U04_072644_1826_XI_02N224W	2022‐01‐24	After	Crater	5.37
CaSSIS	MY36_019001_177	2022‐02‐25	After	Low albedo spots	4.0
HiRISE	ESP_073633_1840	2022‐04‐11	After	Low albedo spots	0.25
HiRISE	ESP_074701_1840	2022‐07‐04	After	Crater	0.25
HiRISE	ESP_074912_1840	2022‐07‐20	After	Low albedo spots	0.25
CaSSIS	MY36_021608_005	2022‐09‐27	After	Crater	4.0
HiRISE	ESP_075901_1840	2022‐10‐05	After	Crater	0.25

### 3.1 CTX and CaSSIS

**Figure 3:**
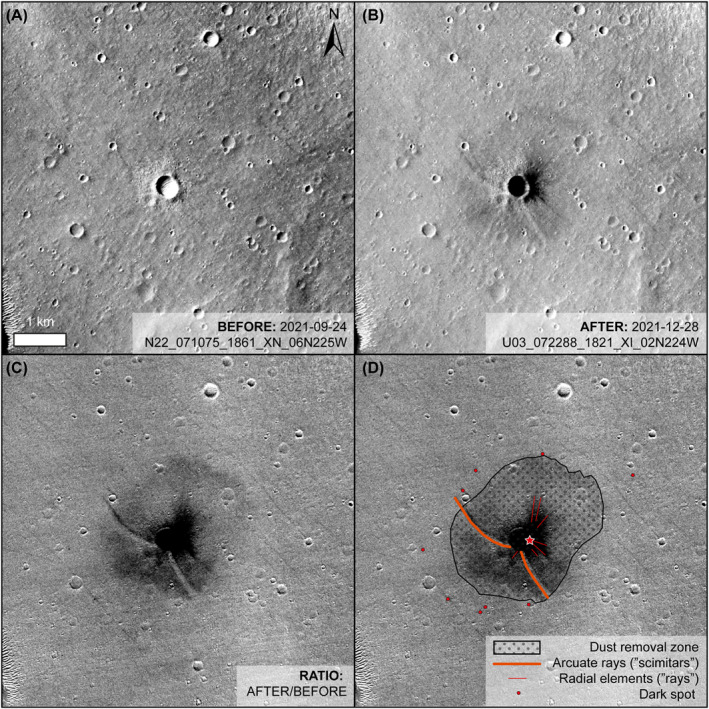
CTX analysis of the S1034a new impact crater (red star), on the rim of a pre‐existing crater. (a) Before the impact. CTX image N22_071075_1861_XN_06N225W, taken 29 days before the impact event. (b) After the impact. CTX image U03_072288_1821_XI_02N224W, taken 66 days after the impact event. (c) Image ratio of before and after images in panels (a, b), highlighting changes at the surface over 95 days, which includes the S1034a impact event. (d) Changes identified in the CTX ratio image, and the location of S1034a impact crater, which was not visible in CTX images.

We used two CTX images (N17_069097_1848_XI_04N225W and N22_071075_1861_XN_06N225W; Figure 3) taken before the impact event, and available through the NASA Planetary Data System (PDS), to make a stereo Digital Terrain Model (DTM) at 20 m/px, and accompanying orthorectified images (orthoimages) at 6 m/px. We produced these data products with standard, well‐validated methods (e.g., Kirk et al., 2003, 2008) using the Integrated Software for Imagers and Spectrometers (ISIS (Adoram‐Kershner et al., 2020)), freely available through the United States Geological Survey (USGS), and the commercial image analysis software SOCET SET, available from BAE Systems. Using the convergence angle of the image pair, image pixel scale, and the Root Mean Square (RMS; 0.4) of the pixel correlations during SOCET SET bundle adjustment (Kirk et al., 2003; Okubo, 2010), we estimate the vertical precision for this DTM to be 2.8 m. We then used this DTM in SOCET SET to co‐register and orthorectify all other CTX images, following other change detection studies on Mars (e.g., Davis et al., 2020), with image IDs given in Table 1. We created image ratios to highlight changes between orthoimages using Quantum GIS (QGIS).

CTX image ratios allowed us to observe a roughly elliptical area of low albedo around the new impact crater that is ∼5.8 km^2^ in area and measuring 2.2 × 3.0 km, with the major axis oriented in a SW‐NE direction, similar to other features at new impact craters termed “halos” (Bart et al., 2019). The image ratios also allowed identification of two distinct high‐albedo arcuate rays, or “scimitars” (Burleigh et al., 2012), that extend 1.4 km NW and 1.0 km SE from the SW edge of the large pre‐existing impact crater. Also visible are low albedo radial elements, or “rays”, that extend between ∼150 and 450 m from the new impact crater, for ∼180° from NE to SW. Several large, ∼15 m diameter, low albedo spots are visible in images taken after the impact event, but spatial resolution of the images precludes confident mapping of all these features. Although the collective center of scimitars, rays, and low albedo spots determined a likely location for the new impact crater, confirmation required later HiRISE imaging, as with several other impact events (e.g., Daubar et al., 2022).

**Figure 4:**
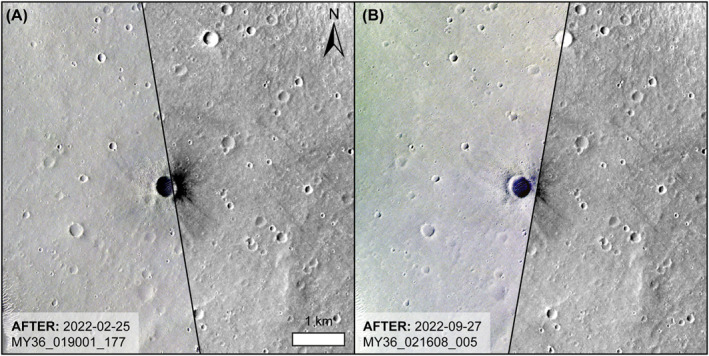
CaSSIS images of the impact area (a) CaSSIS false‐color (NPB) image MY36_019001_177 (left of black line) taken 125 days after the S1034a event. (b) CaSSIS false‐color (NPB) image MY36_021608_005 (left of black line) taken 339 days after the S1034a event. The background in both images is CTX image U03_072288_1821_XI_02N224W.

We used a CaSSIS image (MY36_019001_177; Figure 4) taken soonest (125 days) after the impact event to map further low albedo spot occurrence, and help plan subsequent HiRISE observations. This CaSSIS image was a three‐color product, with band centers (and bandwidths) of BLU = 499.9 (118.0) nm, PAN = 675.0 (229.4) nm, and NIR = 936.7 (113.7) nm (Thomas et al., 2017). We used a false‐color “NPB” combination, corresponding to channels of NIR, PAN, and BLU, respectively (Tornabene et al., 2017) to help identify impact‐related low albedo spots. The impact crater itself was not within this first CaSSIS image, but was covered by a later image (MY36_021608_005), taken 339 days after the impact event. The low albedo region around the impact crater and the scimitars are evident in both images, with the latter image also containing small areas to the east of the pre‐existing impact crater that appear blue in the NPB color product, which correspond to the S1034a impact event.

Due to the varying time between different images, and the likelihood of dust reducing the contrast of new features, we combined all low albedo spot identifications with CTX, CaSSIS, and HiRISE images, as detailed below.

### 3.2 HiRISE

**Figure 5:**
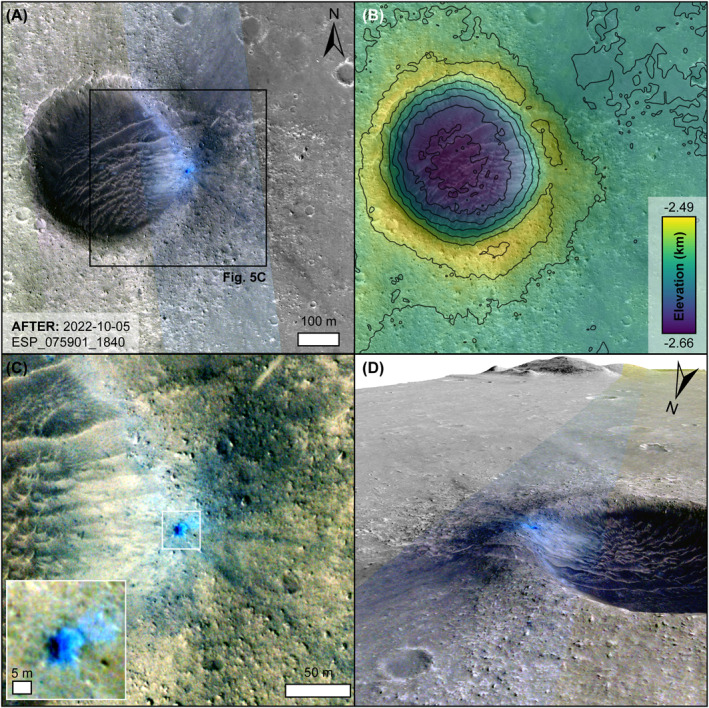
HiRISE image of the new crater. (a) HiRISE image ESP_075901_1840 RED and color (central swath only) centered on the new crater. The black box shows the location of (c). (b) Colorized DTM overlaid on HiRISE RED image ESP_075901_1840. Contours are at 10 m intervals. (c) Detailed view of the new crater in HiRISE color image ESP_075901_1840. Inset shows the detail of the new crater located by the white box. (d) Perspective view of the new crater on the rim of the larger impact crater. HiRISE image ESP_075901_1840 RED and color. Vertical exaggeration is ×2.

We used HiRISE images to investigate the S1034a impact crater and low albedo spots in further detail, using the increased spatial resolution, but limited slightly by the increase in time between the impact event and acquisition of images. We used a stereo DTM (1 m/px) and orthoimages (0.25 m/px), available from the HiRISE PDS node, which used the HiRISE image pairs ESP_074701_1840 and ESP_075901_1840 (Figure 5), with 93 Earth days between these observations. Using the same method as with CTX, the RMS of the pixel correlation (0.47) gives an estimated vertical precision of 0.47 m for this DTM. The S1034a impact crater occurred at an elevation of −2635 m, with the mean difference and standard deviation between this DTM and Mars Orbiter Laser Altimeter of 0.67 and 5.6 m, respectively.

The new impact crater has an irregular, slight elliptical shape, with major and minor axis diameters of 9.7 and 8.3 m, respectively. The rim of the new impact crater is irregular in shape, possibly caused by the presence of boulders. The elongation of the crater occurs in the SW‐NE direction. The quality of the DTM at the pixel scale prohibits a direct depth measurement, but using the crater rim shadow, and assuming a parabolic shape (Chappelow, 2013), we estimate a crater depth of 2.8 ± 0.6 m (assuming one pixel uncertainty). This new impact crater occurs ∼30 m inside and ∼5 m below the eastern rim of a pre‐existing ∼440 m diameter impact crater, which itself has a depth of ∼30 m. This older crater is likely responsible for the large number of boulders on the surface that extend approximately one crater diameter from the rim of the older crater and hamper the search for low albedo spots in this annulus. This pre‐existing crater is likely one of many rocky ejecta craters, indicative of ejection of strong basaltic rock from below an impact generated regolith up to about 20 m thick (Golombek et al., 2017; Warner et al., 2017). Color coverage in HiRISE images inherently occurs in the central swath, with the new crater successfully targeted in image ESP_075901_1840. Despite being limited to ∼1.1 km in width, the HiRISE color information shows that the relative freshness of the new impact crater is evident as appearing blue in false color images. This relatively blue appearance typically indicates that brighter, redder surface dust has been removed in these areas. Both the inside of the new crater, and an area outside the crater to the NE, which is upslope, are noticeably bluer in color than anywhere else in the entire HiRISE image. Also visible are low albedo blue rays, ∼5–10 m in width and up to ∼150 m in length, extending away from the new crater in SE, E, and NE directions. Additionally to the NE, a low albedo blue area ∼60 × 100 m appears to be an area of dust removal that emanates from the new crater.

**Figure 6:**
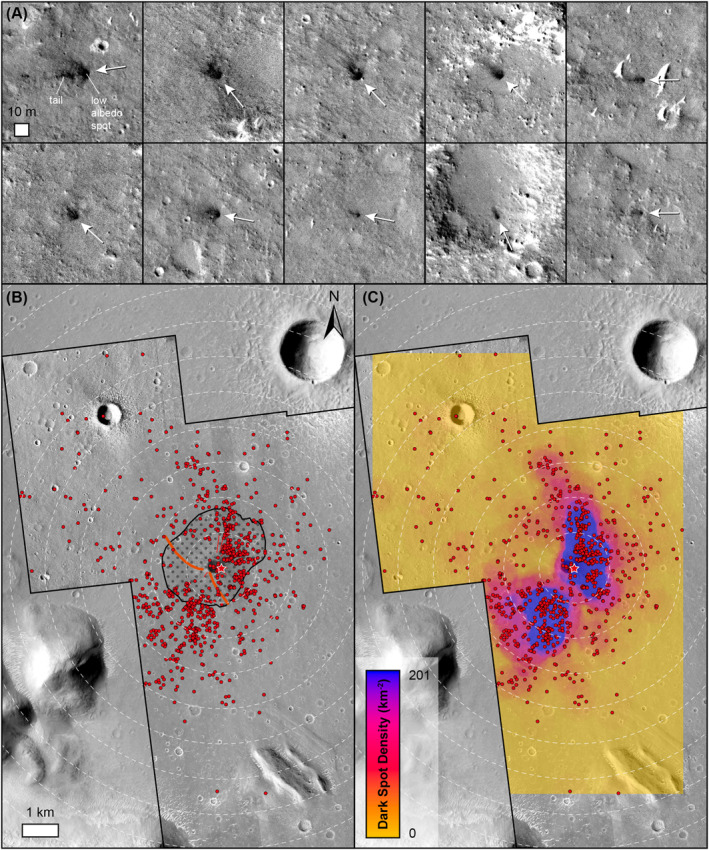
HiRISE low albedo spots and tails. (a) Examples of low albedo spots and associated tails in HiRISE image ESP_073633_1840. Arrows show the inferred approximate direction of impact to create the tail features in each case, with the S1034a impact crater in the opposite direction in each case. (b) Distribution of 910 low albedo spots mapped in three HiRISE images. Red star is the S1034a impact crater. (c) Density of the 910 low albedo spots around the S1034a impact crater.

**Figure 7:**
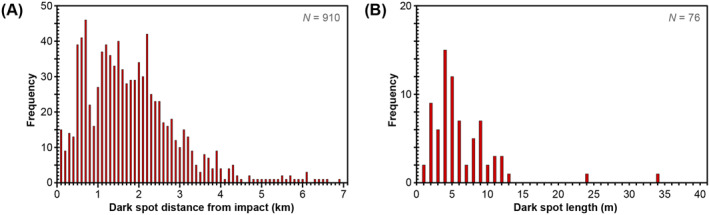
Low albedo spot distance from the new impact crater. (a) Histogram of the distance from the S1034a impact crater of all 910 low albedo spots identified in HiRISE images. (b) Histogram of the tail length of the 75 largest low–albedo spots identified in HiRISE images.

We identified 910 individual low albedo spots surrounding the new impact crater, sometimes with distinctive tails that are oriented away from the new impact crater (Figure 6). We interpret the low albedo tails to be the result of downrange removal of dust using secondary impact processes (e.g., ejecta or blast from the secondary impact). Because of the lack of a HiRISE image before the event, our identification of low albedo spots is limited to those features that we are confident are impact‐related (e.g., not shadows, unrelated to local topography, oriented away from new impact crater), and thus represents a minimum value. No definite resolvable craters were identified within the low albedo spots, meaning that any crater must be less than approximately 1 m in diameter. These low albedo spots are found 51 m to 6.84 km from the new impact crater (∼11–1520 crater radii), with a median distance of 1.58 km (Figure 7). We identified 27 low albedo spots inside the older impact crater, with the remainder found outside. Using a circular neighborhood method of 1 km diameter, we calculated maximum low albedo spot densities of ∼200 km^−2^ occur at distances of up to ∼1.7 km to the NE and ∼2.6 km to the SW of the new impact crater. The areas of maximum low albedo spot densities coincide with the locations of low albedo radial elements identified in CTX image ratios, extending to the NE and SW of the new impact crater. Areas of lower low albedo spot density correspond to the high albedo arcuate rays, or scimitars, in CTX images, extending to the NW and SE of the new impact crater. The size of the low albedo spots is more difficult to determine, with the majority being only several HiRISE pixels in size. Nonetheless, we measured the length of the tails of 76 of the most distinctive low albedo spots in HiRISE image ESP_073633_1840, which ranged from 0.4 to 33.8 m in length, with a median length of 4.5 m. We note a weak negative correlation between distance from the new impact crater and length of low albedo spot tail.

## 4 Discussion

In this section, we discuss the S1034a event in terms of the nature of secondary impact processes, in order to better understand the processes and detection of small new impact events on Mars.

### 4.1 Secondary Impact Processes

The S1034a impact event shares several notable similarities, and some important differences, with other new impacts on Mars. Arcuate rays, or scimitars, are rare at new impact sites on Mars, being present at only 10% of sites (Daubar et al., 2022). These scimitar patterns are likely the result of surface dust redistribution caused by the interaction of shock waves from (a) the projectile in the atmosphere, and (b) the ground impact, rather than seismic wave propagation, and their orientation can be used as an indicator of the direction of the projectile (e.g., Burleigh et al., 2012; Ivanov et al., 2010, 2020). Given that the orientation of the scimitars and the distribution of fresh material (blue in HiRISE color images) both suggest that the bolide direction was from the SW, we can use the height of the surface in that direction to rule out extremely low impact angles. The mound that is 5.34 km to the SW of the impact has a height above the impact surface of 434 m, meaning that the bolide angle had to be greater than 4.6° to avoid impacting into this mound. This assertion is supported by the S1034a impact crater not being highly elliptical, which rules out impact angles less than about 15° (e.g., Collins et al., 2011). This analysis agrees with the apparent absence of a zone of avoidance, which numerical experiments have shown to occur uprange from impacts of small projectiles at angles less than about 20°, with ejecta forming a “butterfly” pattern (e.g., Luo et al., 2022). It is worth noting that the apparent butterfly pattern formed by the low albedo spot density is perpendicular to that formed by a low angle impact. Out of all 910 low albedo spots, 346 are found within the low albedo area (“halo”), of which only 15 occur within the scimitar features. Given that our identification of the low albedo spots requires a secondary impact causing localized dust redistribution, often causing the distinctive tail feature, it is possible that the scimitar‐forming process(es) have either prohibited or masked low albedo spot formation. However, even if the scimitars are likely responsible for the apparent low density of low albedo spots in their neighboring areas, it is still possible that secondary impact craters are present but below the limit of the image resolution for this event.

**Figure 8:**
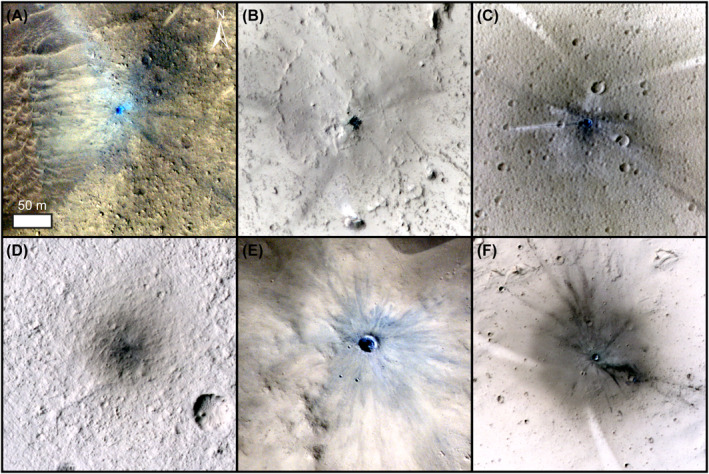
Surface target comparison of the S1034a crater with other small new impact craters with relatively large halos from Bart et al. (2019). (a) The S1034a impact crater, diameter = 9 m. HiRISE image ESP_075901_1840 (centered: 11.36°N, 203.51°E) (b) New impact crater, diameter = 11 m. HiRISE image PSP_007749_1915 (centered: 11.37°N, 203.50°E). (c) New impact crater, diameter = 3.2 m. HiRISE image ESP_035354_1855 (centered: 5.37°N, 26.01°E). (d) New impact crater, diameter = 6.3 m. HiRISE image ESP_037033_2040 (centered: 23.86°N, 265.92°E). (e) New impact crater, diameter = 21.8 m. HiRISE image PSP_004038_2005 (centered: 20.43°N, 3.29°E). (f) New impact crater, diameter = 8.4 m. HiRISE image ESP_034372_1665 (centered: 13.25°S, 200.73°E). All images are HiRISE color with identical scale and orientation.

Superficially, the S1034a impact event appears similar to a new impact crater identified at 13.25°S and 200.7°E in HiRISE image ESP_034372_1665 (Daubar et al., 2016) in the Memnonia region of Mars (Figure 8f). The Memnonia impact crater has a comparable diameter of ∼8.5 m, but with a significant reduction in the size and number of associated secondary impact features. The surrounding halo is only ∼150 m in diameter, rather than the 2.2 × 3.0 km area with the S1034a event. The ratio of halo to crater diameter (289:1) for the S1034a event is also larger than most other halos at new impact sites (Bart et al., 2019). The S1034a halo is an apparent outlier from the general trend given by Bart et al. (2019), and noticeably occurs on a surface target different from four other examples of similar extreme halo sizes (Figure 8). The Memnonia impact crater is also surrounded by low albedo spots with no identifiable impact crater, but only of the order of 100 low albedo spots are present, with roughly a third to a half occurring in distinctive rays or clusters. The maximum distance from a low albedo spot to the primary Memnonia crater is only ∼1.1 km, rather than the 6.8 km for the S1034a event. Finally, the Memnonia crater has scimitars in a SW location similar to S1034a, but with a length of ∼180 m rather than 1.0–1.4 km. Overall, the size of the primary craters of these two events is almost identical, but there is roughly 1–2 orders of magnitude difference between a number of secondary characteristics. The porosity, strength, layering, impact angle and primary impact velocity could all have an effect on ejecta processes responsible for these differences (e.g., Housen & Holsapple, 2011; McEwen & Bierhaus, 2006) but with oblique (e.g., Yamamoto, 2002; Yamamoto et al., 2005) and low porosity (e.g., Holsapple et al., 2002) impacts producing greater volumes and velocities of ejecta, respectively. Given that we can rule out an oblique impact for the S1034a event, it is likely that target porosity is one of the main controlling factors in the large number of secondary features present. The relatively small S1034a impact occurring on the rim of an existing impact crater could provide a coherent low porosity bedrock target. But it is clear that other new craters of a similar size on different surface targets (Figure 8) are also capable of producing large halos, although not commonly (Bart et al., 2019). Given the ubiquitous dust‐rich surface, such a high number of secondary features could be rare for such small impact events on Mars, and could lead to other similar sized events being not identified and under‐reported.

**Figure 9:**
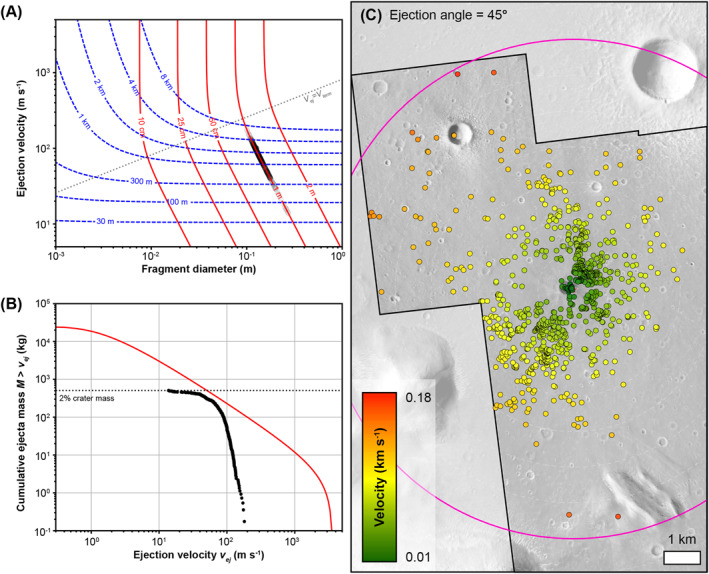
Results of our ballistic modeling incorporating atmospheric drag. (a) Ejecta range (blue dashed line) and resulting crater size (solid red lines) are shown as a function of fragment diameter and ejection velocity. The dotted gray line shows the threshold when ejection velocity equals terminal velocity. (b) The cumulative mass‐velocity distribution of the ejected fragments (black circles) compared to the total ejecta mass‐velocity distribution (solid red line). (c) The impact velocities of the low albedo spot‐forming ejecta, estimated for an ejection angle of 45° in our ballistic model.

The distribution and size of the low albedo spots provide an opportunity to estimate the mass‐velocity distribution of ejecta produced by the S1034a crater event. As neither craters nor boulders are visibly identifiable at any low albedo spot, we assume a maximum crater diameter of 1 m (the smallest resolvable crater in HiRISE images) at each spot to derive an upper bound on ejecta fragment sizes. Applying conventional crater scaling relationships for sand (Housen & Holsapple, 2011), in the absence of an atmosphere, a 1 m secondary crater at 3 km range from the primary would be produced by a ∼10 cm diameter fragment ejected at a speed of approximately 100 m s^−1^. However, at such small fragment sizes, atmospheric drag can be important even in Mars' thin atmosphere. We therefore calculated the range and secondary crater diameter as a function of fragment diameter and ejection speed (Figure 9a) by solving the equations of ballistic motion with atmospheric drag before applying the crater scaling relationships. Our calculations assume a flat planet, a constant gravitational acceleration of 3.71 m s^−1^, and an atmosphere of constant density 0.02 kg m^−3^, all justified by the short ejection distances (<7 km). We also assume an ejection angle of 45°, a spherical fragment of density 2,700 kg m^−3^, a target density of 1,500 kg m^−3^, and a drag coefficient of 1. The appropriate drag coefficient for the ejected fragments will depend on their shape and velocity relative to the ambient sound speed in Mars' atmosphere (Mach number). For simplicity, we adopt a conservative constant drag coefficient of 1, appropriate for angular fragments at low Mach numbers or more spherical fragments at higher Mach numbers (e.g., Loth et al., 2021). Analysis of both orbital images, and in situ geology at the InSight landing site, define a stratigraphy of thin dust over sand‐dominated regolith less than 20 m thick, grading into basaltic breccia and eventually solid basalt (Golombek et al., 2017, 2020a, 2020b; Warner et al., 2017, 2022). It is therefore likely that the new impact occurred in poorly consolidated material, hence, our choice of target density. An important threshold is defined when the ejection speed is equal to the terminal velocity of the fragment in Mars' atmosphere (gray dotted line, Figure 9a). While the effects of drag can be neglected for fragments ejected slower than their terminal velocity, fragments ejected much faster than their terminal velocity are slowed appreciably by the atmosphere and strike the ground much slower and more steeply than they are ejected. In the former case, range (blue dashed line, Figure 9a) depends only on ejection speed and not on fragment size, while crater size (red line, Figure 9a) increases with both ejection speed and fragment diameter. In the latter case, range depends on both ejection speed and fragment diameter, but crater size depends primarily on fragment diameter (as impact speed tends toward terminal velocity).

Our tabulated calculation results allow us to invert the range of each low albedo spot (and assumed 1 m crater diameter) for the diameter and ejection speed of each fragment (black circles, Figure 9a) and hence the mass‐velocity distribution of the ejected fragments (Figure 9b). If the craters at each spot are close to 1 m in diameter, then the implied fragment diameters (10–27 cm) are large enough that their ballistic flights were little affected by atmospheric drag, and ejection speeds (14–180 m s^−1^) were below the terminal velocity. On the other hand, if the craters at the spots are substantially smaller, atmospheric deceleration may have been important. The total mass of ejected fragments is >500 kg, which corresponds to approximately 2% of the primary crater mass, based on its estimated dimensions and a target density of 1,500 kg m^−3^. This relationship is similar to that observed for large craters with secondary craters (e.g., Melosh, 1989), suggesting that the amount of material required to create secondary craters is a small fraction of that removed by the primary crater regardless of size. The cumulative mass‐velocity distribution of the secondary crater‐forming fragments (Figure 9b) is consistent with ejecta scaling relationships for a sand‐like target (Housen & Holsapple, 2011), with most of the remaining ejecta likely residing in the proximal continuous ejecta blanket. The mass of secondary‐forming fragments ejected faster than ∼50 m s^−1^ approximately represents half of all the ejecta launched at these speeds.

### 4.2 Detection Rates

Despite the detection of the seismic event with InSight data, the S1034a impact crater was not identified through standard orbital methods (Daubar et al., 2022; Posiolova et al., 2022). Instead, analysis of indirect evidence in the form of low albedo spots allowed subsequent reanalysis, and targeted imaging was required to positively identify a new impact crater. This process has implications for the analysis of InSight data, in that it is likely that other impact events could also have not yet been identified in orbital images despite the detection of seismic events. The largest new impact crater detected by InSight, the S1094b event, has a diameter of ∼150 m and produced a large number of associated low albedo spots interpreted as the result of secondary impact processes (Dundas et al., 2023; Posiolova et al., 2022). Secondary craters are often identifiable as the source of the low albedo spots for the S1094b event, with diameters typically up to ∼5–10 m corresponding to low albedo spot sizes of ∼100–150 m. If the same scaling is applied to the S1034a event, then we would expect secondary crater diameters to typically be 25 cm, for the mean low albedo spot size of 5 m, or 67 cm, for a larger 10 m low albedo spot size. Both of these secondary crater diameters would be unresolvable in HiRISE images, which matches with our observations. This analysis is important, as the discovery of this new impact crater relied on secondary impact processes. In this case, without being able to identify secondary craters, the low albedo spots become key to identification, and it is necessary to ask how long these features persist.

**Figure 10:**
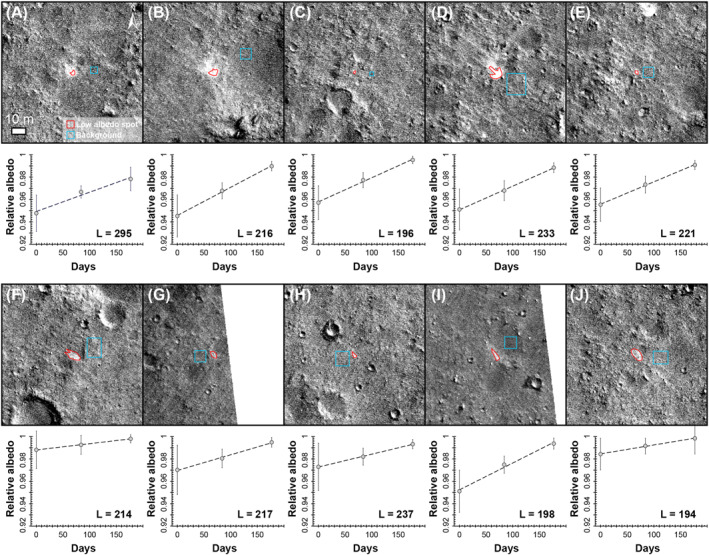
Results of the fading analysis for 10 low albedo spots. In each example (a–h): top is a ratio of the latest HiRISE image (Day 177: ESP_075901_1840) divided by the earliest HiRISE image (Day 0: ESP_073633_1840), with the location of the region of interest used to extract values for the low albedo spots (red) and background (blue) shown; bottom is a plot of the relative albedo over time, with a linear fit used to calculate the lifetime (L) in Earth days of the low albedo spots.

To determine the rate at which the low albedo spots fade over time, we studied 10 low albedo spots that occur in three separate HiRISE images (Day 0: ESP_073633_1840; Day 84: ESP_074701_1840; Day 177: ESP_075901_1840) using the quantitative albedo measurement method of Daubar et al. (2016) (Figure 10). As part of this approach, we identified areas for analysis by creating a ratio of the Day 0 and Day 177 images, which highlighted the fading effect at the low albedo spots. All 10 low albedo spots brighten over time, and show a similar rate of fading, with the time taken to completely fade estimated as between 194 and 295 Earth days, with a median time of 216 days. The first image used in this analysis was taken 170 days after the S1034a impact event, suggesting that if fading occurred in a consistently linear manner, then the low albedo spots would have been almost twice as dark immediately after formation. If a non‐linear function was instead applied, then the low albedo spots could have been even darker, and would fade at a slower rate, as noted for some blast zones around new impact craters on Mars (Daubar et al., 2016). Overall, the rapid rate at which the low albedo spots have faded suggests that there should be no evidence of secondary impact processes at this new impact event within 200–300 days after formation. It is important to note that the low albedo spots in our study formed in a region that was predicted (Golombek et al., 2017), and confirmed (Golombek et al., 2023) to have relatively high dust deposition rates. Given that dust accumulation measured at previous landing sites on Mars varies by at least two orders of magnitude (Lorenz et al., 2021), it is likely that the rate at which similar low albedo spots fade in regions of lower dust deposition will be slower than that observed in our study. This consideration likely explains the difference between the rapidly fading low albedo spots observed in our study, and the slow fading of (much larger) blast zones around new impact craters, which typically take ∼8 Mars years (Daubar et al., 2016). Overall, although dusty areas on Mars help identification of new impact craters through orbital image analysis (Daubar et al., 2022), the evidence for smaller secondary impact features in these high dust areas is masked relatively quickly, meaning that images must be taken within roughly 0.5 Mars years after an impact event for these smaller features to have a higher chance of being detected. This timescale places useful limits on future image searches attempting to identify small new impact craters in dusty areas on Mars.

## 5 Conclusions

One of the goals of the NASA InSight mission was to attempt to detect new impact events on the surface of Mars (Banerdt et al., 2020). By the second year of surface operations, this goal had been met (Garcia et al., 2022), but importantly, discoveries of new impact craters are ongoing (e.g., Daubar et al., 2023). The results of this study are important in current and future attempts to identify new impact events, both with and without seismic data. Our analysis demonstrates that not only can secondary craters be used to discover new impact events but also directional analysis can be used to successfully indicate the location of the primary impact crater. However, the number of secondary craters and associated features (e.g., low albedo spots, tails) is likely related to the target material, with the porosity of the surface at the impact site a key control in determining whether a significant number of secondary features are produced. In addition, at small (∼10 m diameter) craters on Mars, the secondary crater features are obscured by dust on relatively short timescales, with our fading analysis suggesting complete fading in just 200–300 days after formation. Our results place useful limits for future image searches attempting to identify small new impact craters in dusty areas on Mars, and suggest that it is likely that more new impact craters can still be detected in existing data sets.

## Data Availability

InSight seismic data are available via the SEIS data service (InSight Mars, 2019a, 2019b), and the catalog of events through the Marsquake Service (InSight Marsquake, 2023). The HiRISE DTM and orthoimages (McEwen, 2009), and all other HiRISE images (McEwen, 2007) are available at the HiRISE PDS node. CaSSIS images are available through the ESA Planetary Science Archive (PSA (Thomas, 2021)). Our derived CTX DTM and orthoimages, shapefiles and tables of the low albedo spots and new impact crater, and python code for our ballistic ejecta models are available at the accompanying Figshare Archive (Grindrod, 2024). This research has made use of the United States Geological Survey (USGS) Integrated Software for Imagers and Spectrometers (ISIS).
